# Spatiotemporal analysis of electronic cigarette discussion on Twitter/X using natural language processing

**DOI:** 10.1186/s12889-026-27843-x

**Published:** 2026-06-04

**Authors:** Zidian Xie, Jiamu Tang, Dongmei Li

**Affiliations:** 1 Clinical and Translational Science Institute, University of Rochester Medicine, The United State of America, 265 Crittenden Boulevard CU, Rochester, NY 420708, 14642-0708 USA; 2 Goergen Institute for Data Science and Artificial Intelligence, University of Rochester, Rochester, NY USA

**Keywords:** E-cigarettes, Social media, Twitter/X, Deep learning model, Sentiment, Topic

## Abstract

**Background:**

Electronic cigarettes (e-cigarettes) have become popular in recent years, particularly among the youth and young adults. This study aims to examine the spatiotemporal patterns of online discussion of e-cigarettes on Twitter/X.

**Methods:**

Through the Twitter API (Application Programming Interface), over 3 million e-cigarette-related tweets were collected from March 11, 2021, to March 14, 2023, using related keywords, such as “e-cigarette” and “vaping”. After data cleaning (such as removing duplicates and retweets) and filtering, 2,140,439 non-commercial tweets were identified. Two human coders independently hand-coded 300 randomly selected tweets regarding relevance (yes or no), sentiment (positive, negative, or neutral), and whether the Twitter user is a likely e-cigarette user (yes or no). An additional 2,000 randomly selected tweets were single-coded. The labeled 2,300 tweets were used to fine-tune a pre-trained RoBERTa (Robustly Optimized BERT) model, which achieved good performance (F1 scores > 0.7). The Latent Dirichlet Allocation (LDA) method was used to identify the major topics in tweets with either positive or negative sentiment.

**Results:**

We observed a noticeable increase in the number of e-cigarette-related tweets, especially in the UK and Australia, during the study period. Nearly half of the tweets (49.7%, 1,063,317/2,140,439) were neutral. The proportion of tweets with a positive sentiment toward e-cigarettes was higher than that with a negative sentiment, at 27.0% vs. 23.3%. Except for Australia, in the US and UK, especially Canada, there were more positive tweets than negative ones. There was a rising trend in the proportion of tweets with a negative sentiment in the UK and Australia. Additionally, e-cigarette Twitter users were more likely to hold a positive sentiment toward e-cigarettes than non-users, 41.19% vs. 9.74%. Positive topics framed vaping as a desirable, emotionally driven alternative that supports smoking cessation, whereas negative topics emphasized health risks, youth harm, environmental concerns, and calls to quit despite perceived reduced harm.

**Conclusions:**

Online sentiments of e-cigarettes on Twitter varied over time and across different countries. E-cigarette users and non-users held different sentiments toward e-cigarettes. Findings from this study provide timely monitoring in online discussion of e-cigarettes on social media, offering valuable guidance for future tobacco regulations.

**Supplementary Information:**

The online version contains supplementary material available at 10.1186/s12889-026-27843-x.

## Introduction

Electronic cigarettes (e-cigarettes) are the most commonly used tobacco products in US middle and high school students, according to the 2024 National Youth Tobacco Survey [1]. The prevalence of current e-cigarette use (vaping) is 7.8% among high school students and 3.5% among middle school students in 2024 [2]. Although the long-term health effects of vaping are largely unknown, experimental and clinical studies have reported the acute health effects of vaping, including toxicity in the respiratory and cardiovascular systems [3]. Epidemiology studies associated vaping with respiratory disorders, such as wheezing, asthma, and chronic obstructive pulmonary disease, and mental health issues [4–10].

Regulatory policies for e-cigarettes vary across countries. In 2009, the U.S. began regulating e-cigarettes as tobacco products, and in 2016, the US Food and Drug Administration (FDA) expanded its authority to regulate e-cigarettes as tobacco products [11]. In 2020, to prevent youth access to flavored e-cigarettes, the US FDA implemented a flavor enforcement policy to restrict the sale of all unauthorized flavored cartridge-based e-cigarettes other than tobacco and menthol flavors [12]. On June 23, 2022, the US FDA issued marketing denial orders (MDOs) to all JUUL products in the US, which prohibits the company selling and distributing these products in the US market [13]. While the JUUL products were dominant in the US e-cigarette market in 2018 and 2019 [14], these MDOs can lead to active online discussion around the policy change as well as the e-cigarettes. The e-cigarette products in Canada have a nicotine concentration cap of 20 mg/ml, with federal laws restricting their advertising, mandating plain packaging, and prohibiting their sales to minors [15]. In the UK, e-cigarettes are regulated as a less harmful smoking cessation tool with restrictions on nicotine strength (20 mg/ml), tank capacity (2 ml), and advertisement [16]. Australia has the world’s strictest e-cigarette policies, with e-cigarettes classified as prescription-only medicines in 2021 and announced plans in 2023 to ban disposable vapes and restrict flavors to curb youth uptake of e-cigarettes [17]. Regulatory policies on e-cigarettes can have a significant impact on the perception and prevalence of e-cigarette use. For example, the UK government’ 2024 vaping policy announcement exacerbated negative harm perceptions of e-cigarettes [18]. Furthermore, cross-national differences in e-cigarette regulatory policies may shape public perceptions and influence the prevalence and patterns of e-cigarette use.

With the continuing battle between tobacco regulatory policies and aggressive industry marketing, monitoring public discussion of e-cigarettes is critical, as they may directly influence the prevalence of e-cigarette use. Risk perception theory suggests that individuals’ behaviors are influenced not only by objective risk but also by subjective perceptions of harm and benefit [19]. Therefore, it is critical to monitor public discourse of e-cigarettes over time. Tobacco regulatory policies or measures, such as the announcement of e-cigarette flavor ban locally or nationally, can influence public discussions and online sentiments towards e-cigarettes by signaling potential health risks [12, 20–22]. Conversely, industry marketing strategies can reshape public discussions by promoting favorable attitudes toward e-cigarette use [23–26]. Media framing theory highlights how the presentation of information, such as tobacco regulatory policy announcements or product marketing, can influence public discussion and sentiment [27]. Twitter (now X) provides a valuable real-time lens into public discourse since Twitter posts capture immediate public responses to both tobacco regulatory policies (e.g., e-cigarette flavor ban announcements) and industry marketing activities (e.g., e-cigarette promotion by influencers). Therefore, Twitter data have been extensively used to examine online discussions of e-cigarettes, as well as how regulatory policies influence these discussions [20–22, 28, 29]. Previous studies showed that Twitter users’ sentiments toward e-cigarettes became significantly more negative following announcements by New York State and the US FDA regarding bans on the sale of flavored e-cigarettes, excluding menthol and tobacco flavors [21, 22]. Subsequent work further evaluated the impact of New York State’s comprehensive flavor ban—which prohibits the sale of all flavored vapor products except tobacco and other authorized flavors—and found increased prevalence of discussions related to youth, nicotine products, and quitting behaviors using Twitter data from February to November 2020 [20]. Furthermore, one study reported that discussions surrounding vaping cessation became more prominent following the FDA’s announcement and implementation of the flavor enforcement policy [28]. Beyond the U.S. context, one prior study leveraged Twitter data to examine public discussions of e-cigarette prescription policies in Australia and the UK, where e-cigarettes are regulated as medical products [29]. This study found that UK Twitter users expressed more positive attitudes toward the prescription policy than Australian users. Specifically, tweets with positive sentiment in the UK more frequently framed e-cigarettes as a smoking cessation aid, whereas positive tweets in Australia focused on health effects. In contrast, negative sentiments emphasized economic consequences in the UK and concerns about hindering smoking cessation in Australia [29].

Given the rapid evolution of e-cigarette marketing and regulatory policies across countries, it is critical to continuously update and monitor online discussions of e-cigarettes on social media platforms such as Twitter/X across both temporal and geographic dimensions. Using Twitter/X data collected from March 2021 to March 2023, this study examined online discussions of e-cigarettes in the US, Canada, the UK, and Australia using deep learning–based natural language processing approaches. By integrating temporal trends, cross-country comparisons, and user-level e-cigarette use status, this study provides timely evidence to inform the development and evaluation of e-cigarette regulatory policies in the US and other countries.

## Methods

### Data collection

Through Twitter/X streaming API (Application Programming Interface), e-cigarette-related English tweets were collected between May 3, 2021, and March 14, 2023, using keywords directly related to electronic cigarettes, such as “e-cig” and “vape“ [30, 31]. In total, 6,940,065 tweets related to e-cigarettes were collected.

### Data pre-processing

Duplicate tweets and retweets were removed, resulting in 3,045,114 unique tweets. To identify organic discussions, we removed tweets with commercial content (e.g., e-cigarette advertisements from vape shops and small businesses) using keyword filtering within usernames and tweet content (e.g., “deal,” “sale,” “promo”) [20, 22, 32, 33]. This process resulted in 2,575,607 non-commercial tweets. In addition, based on the geolocation information provided by the metadata from the tweets and Twitter/X user accounts, as described in previous studies, we performed geoprocessing to identify tweets from four countries: the United States, the United Kingdom, Australia, and Canada [29, 34].

### Human-guided deep learning language models

In this study, considering some tweets might not be directly related to e-cigarettes even though they contain e-cigarette-related hashtags, first, we determined if the tweet is related to e-cigarettes (Relevancy). Next, we determined the sentiment of each tweet toward e-cigarettes/vaping, including positive, negative, or neutral sentiment. In addition, we determined if the tweet was from a potential e-cigarette user (vapers) or a non-e-cigarette user (non-vapers) depending on whether the tweet contained explicit first-person statements indicating current e-cigarette use. To do this, we manually labeled a random sample of 2,601 tweets to create a reliable dataset for classifier training. Firstly, two human coders independently labelled 300 tweets (randomly selected from the dataset) and set up the codebook. However, the Cohen’s kappa values were below the threshold of 0.70, indicating relatively low inter-rater agreement. Therefore, after resolving the discrepancies among the first 300 tweets through the discussion in the group of three members and the codebook was updated, a second set of 300 tweets was randomly selected and hand-coded independently by the same two coders. For the second round, the kappa value for relevancy reached 0.80, the kappa value for the sentiment achieved 0.72, and the kappa value for the user type (vapers or non-vapers) reached 0.74, indicating substantial inter-rater agreement. Any discrepancy was resolved through a group discussion with three members, and the codebook was further updated. Finally, two trained human coders single-coded an additional 2001 tweets. In total, 2,601 tweets were manually coded.

For classification, in this study we have applied RoBERTa (Robustly Optimized BERT Pretraining Approach), transformed-based models [35]. RoBERTa’s fine-tuning capabilities allow for accurate classification across nuanced categories, making it suitable for sentiment analysis and context-based filtering in social media datasets. We split our labelled dataset (2,601 labelled tweets) into 80% for training and 20% for testing to ensure robust model performance and reliable evaluation. Using RoBERTa’s pre-trained language model, we fine-tuned each classifier for our specific tasks to leverage its advanced language representation on Twitter data. To address imbalances in the dataset, we applied sampling techniques, including oversampling and undersampling, particularly when certain categories (e.g., positive vs. neutral sentiment) were underrepresented. The F1 score is the harmonic mean of precision and recall, offering a balanced model performance measure on imbalanced datasets. Regarding e-cigarette relevance, the model’s F1 score reached 0.87. For sentiment towards e-cigarettes, the model achieved an F1 score of 0.82. The model’s F1 score for identifying vapers was 0.81. Therefore, the RoBERTa models demonstrated good model performance. These models were used to label all non-commercial tweets. Finally, only 2,140,439 tweets were identified as relevant to e-cigarettes.

Given the complexity of determining user identity from an individual tweet, a two-step classification process was employed to enhance accuracy and robustness. First, a RoBERTa-based user type classification model was applied at the tweet level to determine whether a given tweet was posted by an e-cigarette user (vaper). Second, user IDs were aggregated, allowing multiple tweets from the same user to be analyzed collectively. This aggregation ensured a more reliable classification of individual users as vapers or non-vapers, rather than making inferences based on isolated tweets.

### Topic modeling

To uncover prominent themes within public discourse on e-cigarettes, we conducted topic modeling using Latent Dirichlet Allocation (LDA), a generative probabilistic model well-suited for large text datasets [36]. LDA identifies latent topics within a corpus by clustering terms based on co-occurrence patterns, making it highly effective for grouping related themes within tweets that express complex sentiments. Its flexibility and effectiveness in distinguishing nuanced topics across different text samples made it an ideal choice for exploring prevalent themes in our dataset. To ensure consistent and meaningful topic extraction, we standardized the dataset through several preprocessing steps. We converted all text to lowercase, lemmatized terms, and removed stop words, such as personal pronouns and prepositions, using spaCy and the Natural Language Toolkit (NLTK). This preprocessing was crucial for minimizing noise and maximizing coherence within each topic. Additionally, we identified frequent bigrams (e.g., “quit vaping”) and trigrams (e.g., “vaping health risks”) using the Gensim package, treating these common phrases as single terms within the LDA model. This setup enhanced the model’s ability to detect clear, contextually relevant topics. To determine the optimal number of topics, we calculated coherence scores for different numbers of topics and selected the one with the highest score. The coherence score quantitatively evaluates the interpretability of topics, ensuring that each topic represents a distinct theme within the data. Since tweets with a neutral sentiment typically consisted of informational content (e.g., policy updates), topic modeling was restricted to tweets with positive or negative sentiment to better capture opinion-driven discourse related to e-cigarettes. From tweets with a positive sentiment, we identified four main topics, including (1) Vaping helps with smoking cessation, which primarily deals with people discussing their experiences using vaping as a method to quit smoking; (2) Vaping is a better choice than smoking, which focuses on advocating vaping over traditional smoking, highlighting the benefits of e-cigarettes including health and social aspects, often with an advocacy tone against misconceptions; (3) The desire for vaping, which captures tweets about the habitual nature of vaping and its emotional implications; (4) Emotional triggers and routine use of vaping, which highlights the consumerism aspect of vaping, focusing on desires for new products (Supplemental Table 1). There were nine main topics in tweets with a negative sentiment, including (1) Complaints about tobacco industry, which discusses negative sentiments toward big corporations and their influence over the vaping industry; (2) Health risks of vaping, which discusses the severe health risks associated with vaping, particularly focusing on lung damage, addiction, and the inclusion of harmful chemicals; (3) Bad experiences with vaping, which captures the challenges and experiences related to quitting smoking and switching to or from vaping; (4) Irresponsible vaping behaviors in public and youth settings, which discusses the social impact of vaping, especially in inappropriate contexts like schools or around children, often highlighting irresponsible behavior; (5) Vaping is bad for youth, which concerns about the impact of vaping on youth, highlighting health risks and the prevalence of vaping in school environment; (6) Vaping is safer than smoking but not risk-free, which compares the harm of vaping to traditional smoking, arguing that it’s a lesser evil, yet still acknowledging its risks; (7) Urge to quit vaping due to health risks and addiction, which emphasizes the urgent need to quit vaping due to its association with serious health issues like cancer and addiction; (8) Calls for quitting vaping due to health and social perceptions, which discusses the social judgments and stigmas associated with vaping, comparing it to other substance use like drinking or drug use; (9) Dislike vaping, especially the waste coming from disposable vaping products, which captures negative opinions about vaping. It focuses on individuals expressing their dislike for the habit and calling it a bad influence, particularly the growth of disposable vaping products and their implications (Supplemental Table 1).

### Interrupted time series analysis

To evaluate how the FDA JUUL ban announcement might influence the sentiment and Twitter user composition, we conducted interrupted time series (ITS) analysis using segmented regression models. To do this, monthly proportions of tweets with either positive or negative sentiment, as well as the proportion of vapers and non-vapers were modeled as the function of time, the intervention (June 2022), and time after the intervention. To account for potential autocorrelation in the time series data, we fitted autoregressive error models using a first-order autoregressive process, AR (1), within a generalized least squares framework.

## Results

### Identification of e-cigarette-related tweets

**Fig. 1:**
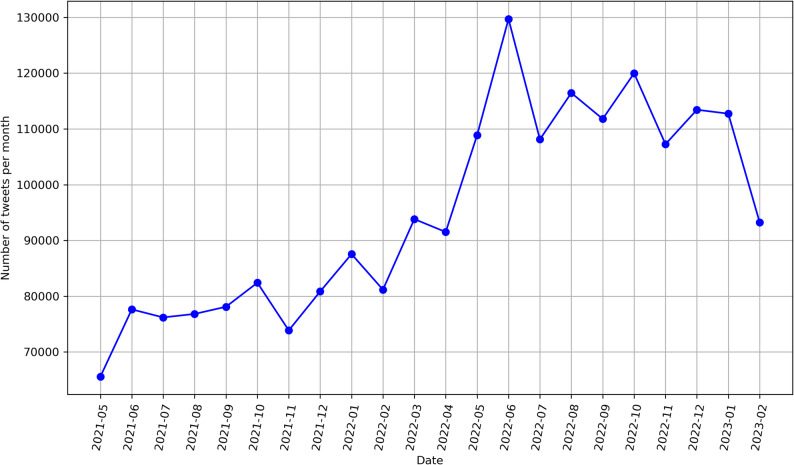
Temporal trend in the prevalence of e-cigarette-related English tweets

From May 3, 2021, to March 14, 2023, we have identified a total of 6,940,065 English tweets using keywords related to e-cigarettes. Among them, there were 2,140,439 unique non-commercial tweets related to e-cigarettes. As shown in Fig. 1, the number of tweets per month exhibited a steady increase over time in our studying period with a notable peak in June 2022 and a slight decrease in February 2023. Based on the geolocation information shared on the tweets, we have identified 430,929 tweets from the US, 90,811tweets from the UK, 43,453 tweets from Australia, and 39,552 from Canada. The rest of tweets were either from other countries or no valid geolocation information. Similarly, the mentions of e-cigarettes on Twitter showed an overall increasing trend in the US, UK, and Australia except in Canada (Supplemental Fig. 1).

### Online sentiments toward e-cigarettes on Twitter/X

Sentiment analysis was conducted to classify tweets into positive, negative, or neutral sentiment towards e-cigarettes. Among the 2,140,439 tweets related to e-cigarettes, 27.00% (578,016 tweets) expressed a positive sentiment towards e-cigarettes, 49.68% (1,063,317 tweets) were categorized as neutral, and 23.32% (499,106 tweets) expressed a negative sentiment (Table 1). While the proportion of tweets with the positive and negative sentiment towards e-cigarettes were similar in the US and UK, the proportion of tweets with a positive sentiment was higher than those with a negative sentiment in Canada, 29.09% vs. 23.94% (P-value < 0.01). In contrast, the proportion of tweets with a negative sentiment was higher than those with a positive sentiment in Australia, 30.39% vs. 23.76% (P-value < 0.01).

**Table 1:** Online perception of e-cigarettes between different user groups

	Overall			Vapers			Non-vapers		
	Pos	Neg	Neural	Pos	Neg	Neural	Pos	Neg	Neural
All (vapers: 603,600; non-vapers: 450,255)	27.00%	23.32%	49.68%	41.19%	11.85%	46.96%	9.74%	37.28%	52.98%
US (vapers: 125,285; non-vapers: 93,341)	26.20%	25.46%	48.34%	40.7%	12.5%	46.9%	10.2%	39.9%	50.0%
UK (vapers: 23,785; non-vapers: 21,121)	27.93%	27.29%	44.78%	46.5%	12.2%	41.3%	11.0%	41.0%	48.0%
Canada (vapers: 10,856; non-vapers: 8,384)	29.09%	23.84%	47.07%	45.6%	10.5%	43.9%	11.4%	38.2%	50.4%
Australia (vapers: 9,187; non-vapers: 8,667)	23.76%	30.39%	45.85%	43.6%	12.4%	44.0%	10.6%	42.3%	47.1%

*Pos* positive, *Neg* negative

**Fig. 2:**
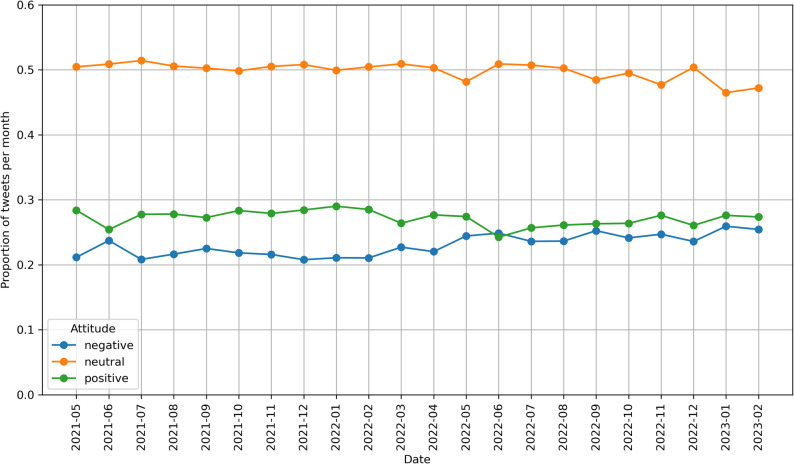
Relative prevalence of tweets with different sentiments toward e-cigarettes over time

As shown in Fig. 2, the relative prevalence of tweets with different sentiments remains relatively constant during the studying period, except the proportion of tweets with the positive sentiment showing significantly decrease (P value < 0.001) immediately after June 2022 followed by the subsequent upward trend (P value = 0.097) while a marginal immediate increase (P value = 0.070) for negative tweets in June 2022 with no significant change in the trend afterwards (P value = 0.597) based on interrupted time series analysis. In addition, the difference in the prevalence of tweets between the positive and negative sentiment became smaller since June 2022. We observed similar trends in the US and Canada (Supplemental Fig. 2). However, in the UK, while there were more tweets with a positive sentiment than those with a negative sentiment before June 2022, the proportion of tweets with a positive sentiment became less than those with a negative sentiment after June 2022. ITS analysis showed a significant immediate increase (P value = 0.021) in the proportion of negative tweets following June 2022, followed by a subsequent decreasing trend (P value = 0.048). In contrast, no significant changes were observed in positive sentiment. In Australia, the number of tweets with either a positive or negative sentiment were similar before June 2022. However, there were more tweets with a negative sentiment than those with a positive sentiment after June 2022. ITS analysis indicated that there was a significant immediate decrease (P value < 0.001) in the proportion of positive tweets following June 2022 while the proportion of negative tweets showed a non-significant increase (P value = 0.135) in June 2022.

### Online discussion of e-cigarettes between vapers and non-vapers

**Fig. 3:**
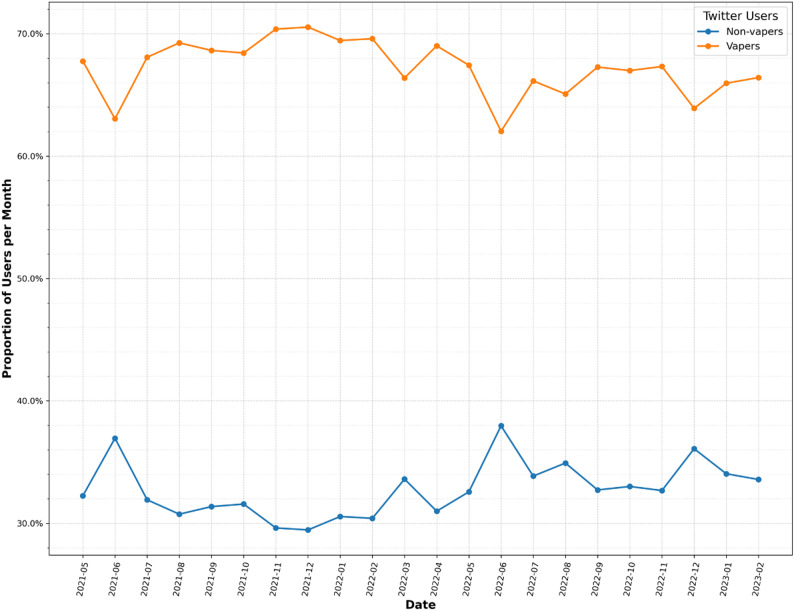
Relative prevalence of vapers and non-vapers among Twitter users posting e-cigarette-related tweets

Among e-cigarette-related tweets, 603,600 unique Twitter users (57.28%) were identified as potential e-cigarette users (vapers), and 450,255 users (42.72%) were categorized as non-e-cigarette users (non-vapers). As shown in Fig. 3, among Twitter users posting e-cigarette-related tweets, the relative prevalence of vapers was dominant over time, ranging from 60% to 70%. Furthermore, the prevalence of vapers showed a slight decrease over time, which reached the lowest level in June 2022. ITS analysis indicated that there was a significant immediate increase (P value = 0.016) in the proportion of non-vapers and a corresponding decrease (P value = 0.022) in vapers following June 2022, but no significant trend changes afterwards. While the relative prevalence of vapers remains constant in the US and Canada, the relative abundance of vapers showed a clear decreasing trend over time in the UK and Australia (Supplemental Fig. 3).

By examining online sentiments of e-cigarettes on Twitter/X, vapers were more likely to exhibit a positive sentiment than non-vapers, 41.19% vs. 9.74% (Table 1). In contrast, they were less likely to hold a negative sentiment than non-vapers, 11.85% vs. 37.28%. By comparison, the proportion of tweets with a positive sentiment was the highest in Canada (29.09%), followed by the UK (27.93%), US (26.20%), and Australia (23.75%). In addition, Twitter users from Australia were more likely to hold a negative sentiment towards e-cigarettes (30.39%), followed by the UK (27.29%), US (25.46%), and Canada (23.84%). We observed similar patterns for vapers and non-vapers between these countries (Table 1).

### Online discussion about e-cigarettes on Twitter/X

To further understand the potential underlying reasons for different sentiments toward e-cigarettes on Twitter/X, main topics were identified from tweets with an either positive or negative sentiment (Supplemental Table 1). As shown in Table 2, four main topics have been identified from e-cigarette-related tweets with a positive sentiment. Two most popular topics included topic 3 “The desire for vaping” (32.77%) and topic 4 “Emotional triggers and routine user of e-cigarettes” (32.93%). Among tweets with a positive sentiment, 22.79% of them held a notion that vaping can help with smoking cessation, and 11.50% perceived vaping as a better choice than smoking. Compared to other three countries, positive tweets from the US were less likely to perceive e-cigarettes as a better choice than smoking or helping with smoking cessation (Table 2). Among nine main topics identified from tweets with a negative sentiment, topic 7 “Urge to quit vaping due to health risks and addiction” was the most popular one (22.18%), followed by topic 4 “Irresponsible vaping behaviors in public and youth settings” (18.08%) and topic 3 “Bad experiences with vaping” (16.24%). Some tweets (8.14%) acknowledged that e-cigarettes are not risk-free. Negative tweets from Australia were more likely to perceive vaping being bad for youth (15.81% vs. 8.06%) and e-cigarettes not being risk-free (19.98% vs. 8.14%), but less likely to concern its environmental impact (6.20% vs. 10.27%) than the overall.

By examining temporal trends in the proportion of topics with either a positive or negative sentiment, among tweets with a positive sentiment, topic 2 “Vaping is a better choice than smoking” showed a peak while topic 4 “Emotional triggers and routine use of e-cigarettes” showed a decrease in June 2022 (Supplemental Fig. 4). At the same time, we observed that topic 6 “Vaping is safer than smoking but not risk-free” showed an obvious peak and topic 4 “Irresponsible vaping behaviors in public and youth settings” showed a decrease in June 2022 among tweets with a negative sentiment (Supplemental Fig. 5).

**Table 2:** Main topics identified in e-cigarette-related tweets with a positive or negative sentiment

Sentiment	Topic	All	US	UK	Canada	Australia
Positive	Topic 1: Vaping helps with smoking cessation	131,754 (22.79%)	26,331 (26.58%)	8,023 (34.19%)	3,110 (29.48%)	2,591 (27.46%)
	Topic 2: Vaping is a better choice than smoking	66,479 (11.50%)	13,670 (13.80%)	3,986 (16.99%)	1,777 (16.84%)	2,221 (23.54%)
	Topic 3: The desire for vaping	189,439 (32.77%)	33,835 (34.16%)	5,780 (24.63%)	3,002 (28.45%)	2,454 (26.01%)
	Topic 4: Emotional triggers and routine use of e-cigarettes	190,339 (32.93%)	25,208 (25.45%)	5,675 (24.19%)	2,661 (25.22%)	2,169 (22.99%)
	Total	578,016 (100%)	99,044 (100%)	23,464 (100%)	10,550 (100%)	9,435 (100%)
Negative	Topic 1: Complaints about tobacco industry	14,482 (2.90%)	2,309 (2.40%)	372 (1.62%)	173 (2.02%)	207 (1.68%)
	Topic 2: Health risks of vaping	22,288 (4.47%)	4,693 (4.88%)	1,032 (4.51%)	467 (5.46%)	721 (5.86%)
	Topic 3: Bad experiences with vaping	81,075 (16.24%)	16,310 (16.95%)	3,915 (17.10%)	1,388 (16.24%)	1,752 (14.24%)
	Topic 4: Irresponsible vaping behaviors in public and youth settings	90,251 (18.08%)	16,082 (16.71%)	3,229 (14.10%)	1,284 (15.02%)	1,385 (11.26%)
	Topic 5: Vaping is bad for youth	40,223 (8.06%)	9,027 (9.38%)	2,399 (10.48%)	864 (10.11%)	1,945 (15.81%)
	Topic 6: Vaping is safer than smoking but not risk-free	40,627 (8.14%)	10,371 (10.78%)	2,622 (11.45%)	1,071 (12.53%)	2,458 (19.98%)
	Topic 7: Urge to quit vaping due to health risks and addiction	110,683 (22.18%)	18,410 (19.13%)	4,766 (20.82%)	1,563 (18.28%)	1,928 (15.67%)
	Topic 8: Calls for quitting vaping due to health and social perceptions	48,212 (9.66%)	9,354 (9.72%)	2,224 (9.71%)	971 (11.36%)	1,146 (9.31%)
	Topic 9: Dislike vaping, especially the waste coming from disposable vaping products.	51,258 (10.27%)	9,664 (10.04%)	2,336 (10.20%)	768 (8.98%)	763 (6.20%)
	Total	499,106 (100%)	96,220 (100%)	22,895 (100%)	8,549 (100%)	12,305 (100%)

## Discussion

In this study, by examining e-cigarette-related posts on Twitter/X, we provided a comprehensive understanding of online discussions of e-cigarettes temporally and geographically. While there was an overall increase in the mentions of e-cigarettes on Twitter/X during the study period, there was an obvious peak in June 2022, especially in the US, which corresponds with the announcement of JUUL ban in the US. Although the proportion of tweets with a positive sentiment was slightly higher than those with a negative sentiment overall, the proportion of tweets with a positive sentiment showed a gradual decreasing trend, especially in the UK and Australia. Further analysis of e-cigarette-related tweets with either positive or negative sentiments, we have identified several main topics, which provide important insights about potential reasons underlying different sentiments.

We observed a gradual upward trend in discussing e-cigarettes on Twitter/X during our study period with a significant spike occurred in June 2022, especially in the US, which coincides with major JUUL-related policy decisions in the United States [13, 14]. These findings indicate that discussions on e-cigarettes might be event-driven, with major regulatory actions and policy debates serving as potential catalysts for increased public discourse.

In this study, nearly half of tweets did not show clear sentiment (labelled as neutral) towards e-cigarettes, they consist of informational-based content, such as policy updates, scientific studies, and product-related announcements, without strong personal opinions. The overall proportion of e-cigarette-related tweets with a positive sentiment was slightly higher than those with a negative sentiment towards e-cigarettes, 27.00% vs. 23.32%. By comparison, tweets from Canada were more likely to show a positive sentiment towards e-cigarettes than those from Australia, 29.09% vs. 23.76%. While the underlying reasons remain elusive, different regulatory policies and cultures in different countries might partly account for such differences [37]. Compared to Canada, Australia has a much more restrictive regulation on e-cigarettes, such as classifying e-cigarettes as prescription-only therapeutic products, which might lead to reduced acceptance and less favorable attitudes toward e-cigarettes. In contrast, the number of tweets with either a positive or negative sentiment was similar in either the US or the UK. This result suggests that the discourse of e-cigarette perception in the US and UK is relatively balanced, with strong advocacy for vaping as a harm reduction tool coexisting with concerns over youth vaping, addiction, and health concerns. Furthermore, as expected, we showed that vapers were more likely to show a positive sentiment towards e-cigarettes than non-vapers on Twitter, which is similar across four countries. This divergence highlights that while vapers tend to frame e-cigarettes in a more favorable light, non-vapers are more likely to view vaping through a public health risk lens, particularly in countries with stringent policies. The polarization of opinions between vapers and non-vapers underscores the complexity of the e-cigarette debate, with strong advocacy on both sides of the discourse.

Our temporal trend showed that while the proportion of tweets with different sentiments remain relatively consistent during our studying period, the difference in relative abundance between tweets with a positive and negative sentiment became smaller since June 2022. From a communication and behavioral science perspective, such convergence may reflect shifting public interpretations of e-cigarette risks and benefits in response to salient policy events. It seems that the proportion of tweets expressing negative sentiment increased over time even not statistically significant, while the proportion of positive tweets showed a significant immediate decline following June 2022, which may be associated with the JUUL ban announcement. Our results further showed an increase in tweets posted by non-vapers in June 2022, suggesting that the JUUL announcement stimulated broader public discussion, particularly among non-vapers, regarding the health effects of e-cigarettes. Notably, in the UK and Australia, the proportion of tweets with positive sentiment decreased, while that with negative sentiment increased, starting from June 2022. Our results showed that the JUUL MDOs in the US was temporally associated with immediate changes in sentiment in the UK and Australia in June 2022. This suggests that tobacco regulatory decisions in one country might influence global public discourse, leading to a significant shift in public discussion of e-cigarettes. Overall, the dynamic nature of vaping discourse underscores the ongoing public debate surrounding its benefits, risks, and societal implications.

Among tweets with a positive sentiment toward e-cigarettes, besides most tweets showed a desire of vaping, nearly a quarter of tweets considered vaping as a smoking cessation strategy, which is more pronounced in the UK (34.19%). From a behavioral science perspective, these themes may reflect “sustain talk” (continued use) and “change talk” (movement toward smoking cessation), respectively. In the UK, e-cigarettes are regarded as an effective smoking cessation method and the medical products [38–40]. About one tenth of tweets considered vaping as a safer alternative to smoking, which was much higher in Australia (23.54%). Therefore, our results suggest that e-cigarettes are more likely to be considered as a safer alternative and effective smoking cessation method in the UK and Australia than the US and Canada, which is consistent with previous findings [41, 42]. Temporal trend showed that there was a peak in topic 2 “Vaping is a better choice than smoking” while there was a decrease in topic 4 “Emotional triggers and routine use of vaping” in June 2022, especially in the US. From a communication perspective, this shift may reflect competing narratives potentially triggered by the announcement of JUUL MDOs in the US. The JUUL ban triggered surges in online discourse about e-cigarettes, with one side of the voice against the ban by emphasizing the benefits of vaping and the desire for e-cigarettes. These narratives may also indicate defensive or justificatory discourse among Twitter users.

Common discussions for the negative sentiment towards e-cigarettes include health concerns of vaping, complaining about vaping in public settings, especially around schools. The topic 4, “Irresponsible Vaping Behaviors in Public and Youth Settings,” was relatively low in Australia, which might reflect the influence of Australia’s much stricter policy on school vaping [43]. From a behavioral perspective, such discussions may represent social norm enforcement and collective concern about youth exposure, which are important drivers of public health behavior. Interestingly, we observed that this topic was significantly lower in June 2022, potentially indicating a shift in attention away from behavioral concerns toward broader risk-related narratives. Recognizing that e-cigarettes are not risk-free, topic 6 experienced significant spikes with the announcement of the FDA’s JUUL ban in June 2022. This pattern aligns with risk communication theory, whereby regulatory actions serve as signals that heighten perceived harm and uncertainty. In addition, the prevalence of this topic was higher in Australia than in other countries, suggesting that e-cigarettes are more likely to be considered not risk-free in Australia. A region-specific discussion emerged around “Environmental Concerns Over Disposable Vapes”, particularly in the UK. Parliamentary debates in 2022 on banning single-use disposable e-cigarettes fueled public discourse about their ecological impact. Such findings highlight the role of communication processes in directing attention to specific dimensions of a health issue, thereby influencing how it is perceived and discussed. While this topic was prominent in the UK, similar discussions were less prevalent in other countries.

In this study, we characterized the spatiotemporal patterns of online discussions about e-cigarettes, providing insight into how Twitter/X users perceived e-cigarettes. Although tweets expressing negative attitude may reflect prevention- or intervention-oriented discourse, engagement with health communication messages is critical for the effectiveness of behavior change interventions [44–46]. Social media engagement is a multidimensional construct that extends beyond content characteristics alone. In digital health contexts, engagement may reflect cognitive, emotional, and behavioral responses to the messages, which is commonly indicated by observable interactions such as likes, shares, and comments. These metrics may capture how users respond to, amplify, and circulate e-cigarette-related content. Prior studies showed that pro-vaping messages were not only prevalent on social media but also received higher levels of engagement (e.g., likes and views) than anti-vaping messages [47, 48]. Therefore, future studies should examine how to design effective health communication messages that achieve high engagement and resonate in online public discourse, thereby potentially promoting healthier behaviors.

This study has several limitations. First, the demographics of Twitter users differ from those of the general population, which may introduce selection bias and limit the generalizability of our findings. As a result, the sentiments and topics identified in this study may not fully reflect public discussion in the broader population. Second, although our deep learning language models demonstrated good performance, they are not perfectly accurate. For example, the sarcasm, slang, or ambiguous language may lead to misclassification and introduce measurement bias to sentiment classification. The short text of tweet could affect the accuracy and interpretability of topic modeling. Third, given the lack of demographic data on Twitter users, we cannot determine the sentiments of e-cigarettes across different demographic groups (e.g., age, sex, and socioeconomic status), especially among youth who are disproportionately affected by e-cigarette use. Future studies should utilize other data sources or methodologies to fill in this gap. Fourth, there may be tweets from social bots, which could introduce noise into our results. In addition, keywords used for collecting e-cigarette-related tweets in this study might not fully represent the e-cigarette-related terms used on social media, which could potentially introduce biases. The change in Twitter’s ownership in 2022 may have influenced the nature of online discourse on the platform, which could have potentially affected the composition and characteristics of the data collected during the study period (2021–2023). Lastly, amid the ongoing battle between the e-cigarette industry and regulatory policy, public perception of e-cigarettes is evolving, requiring ongoing monitoring and timely updates.

## Conclusions

By analyzing millions of e-cigarette-related tweets, this study provided temporal trends and geographic comparisons on online discussion of e-cigarettes. Public discussion of e-cigarettes is evolving, especially with the development of tobacco regulatory policies. While online sentiments of e-cigarettes were similar across countries, some differences persisted, which might be driven by differences in tobacco regulatory policies and societal perceptions. More importantly, what we learned from one country about the impact of certain regulatory policies can provide valuable insights into the potential impact of similar policies in other countries. Health communication frameworks emphasize the role of messaging in shaping public perception, particularly in the context of emerging products such as e-cigarettes [49]. Therefore, while there are different voices about e-cigarettes, it is critical to effectively communicate with the public about the potential health effects of e-cigarettes to further protect public health, especially among the youth.

## Supplementary Information

Supplementary Material 1

Supplementary Material 2

## Data Availability

Data will be made available on individual request.

## Abbreviations

API: Application Programming Interface
E-cigarette: Electronic cigarette
FDA: US Food and Drug Administration
LDA: Latent Dirichlet Allocation
RoBERTa: Robustly Optimized Bidirectional Encoder Representations from Transformers
MDOs: Marketing denial orders
ITS: Interrupted time series
