# Supplementary material for: Spatiotemporal analysis of electronic cigarette discussion on Twitter/X using natural language processing
Source: BMC Public Health. 2026 Jun 4;26:2302. doi: 10.1186/s12889-026-27843-x (PMC13450413; doi:10.1186/s12889-026-27843-x)
Supplement: Supplementary file 1 [file 12889_2026_27843_MOESM1_ESM.docx]

**Supplementary Information**

**Fig S1. Temporal trend in the prevalence of e-cigarette-related tweets in four countries.**

**Figure S2. Temporal trend in the proportion of tweets with different sentiments toward e-cigarettes in four countries.**

**Figure S3. Relative prevalence of vapers and non-vapers among Twitter users posting e-cigarette-related tweets in four countries.**

**Figure S4. The relative prevalence of main topics in tweets with a positive sentiment.**

**Figure S5. The relative prevalence of main topics in tweets with a negative sentiment.**
