# Supplementary material for: Spatiotemporal analysis of electronic cigarette discussion on Twitter/X using natural language processing
Source: BMC Public Health. 2026 Jun 4;26:2302. doi: 10.1186/s12889-026-27843-x (PMC13450413; doi:10.1186/s12889-026-27843-x)
Supplement: Supplementary file 2 [file 12889_2026_27843_MOESM2_ESM.docx]

**Supplemental Table 1. Main topics identified in e-cigarette-related tweets with an either positive or negative sentiment.**

| **Attitude** | **Topic** | **Description** | **Top keywords** | **Example** |
| --- | --- | --- | --- | --- |
| Positive | Topic 1: Vaping helps with smoking cessation | This topic primarily deals with people discussing their experiences using vaping as a method to quit smoking. | vape, smoke, quit, nicotine, try, well, year, help, get, day | I smoked for 50 years. I've had sinus issues for nearly that many years. I'm here to say no more issues once I switched to nicotine vaping. Taste returned and the stink that coated me is no more. I breath better and don't hack up crap every morning. No whizzing at night. Blessed!  well !! i quit smoking an got a vape am feelin good |
|  | Topic 2: Vaping is a better choice than smoking | This topic focuses on advocating vaping over traditional smoking, highlighting its benefits including health and social aspects, often with an advocacy tone against misconceptions. | vape, people, smoking, make, tobacco, cbd, big, fact, great, help | I gave up smoking because of safer products like e-cigarettes - as have people I'm close to. Making changes to combat misperceptions about the risk of vaping, as well as making other alternatives more widely recognised, is really important to me. |
|  | Topic 3: The desire for vaping | This topic captures tweets about the habitual nature of vaping and its emotional implications. | vape, go, get, hit, m, day, buy, time, lose, take | I want to vape.  I need a juul. Can anyone help? |
|  | Topic 4: Emotional triggers and routine use of vaping | This topic highlights the consumerism aspect of vaping, focusing on desires for new products. | vape, get, need, new, buy, love, pen, good, want, flavor | I think i will buy a vape I really need one.  Im so happy i got new vapes. |
| Negative | Topic 1: Complaints about tobacco industry | This topic discusses negative sentiments toward big corporations and their influence over the vaping industry. | vape, bitch, talk, re, big, get, m, know, smoke, hell | well know vapes evil thanks big tobacco.  everytime see someone vaping makes sad evil tobacco industry successfully revitalized |
|  | Topic 2: Health risks of vaping | Discusses the severe health risks associated with vaping, particularly focusing on lung damage, addiction, and the inclusion of harmful chemicals. | vape, lung, nicotine, cause, kill, chemical, addiction, year, inhale, ago | Vaping is bad you can get lung cancer from vaping don't Vape it's not worth it vaping can kill you it can also lead to chromium and nickel and lead in your lungs don't Vape take a stand on vaping. |
|  | Topic 3: Bad experiences with vaping | Captures the challenges and experiences related to quitting smoking and switching to or from vaping. | vape, quit, get, day, try, feel, smoke, bad, smoking, stop | Took me months to burn through that vape juice, I sickened myself with it so much I actually started smoking again. Horrible times, hate it! |
|  | Topic 4: Irresponsible vaping behaviors in public and youth settings | Discusses the social impact of vaping, especially in inappropriate contexts like schools or around children, often highlighting irresponsible behavior. | vape, get, hit, go, kid, shit, f***, see, school, girl | Rehab went to the zoo today and I had fun but it was so embarrassing to be around everyone who went because they were vaping in animal enclosures and swearing really loud in front of little kids and kept getting dirty looks  I was using the bathroom in school and a bunch of girls walked in and started vaping and doing tiktoks what a society |
|  | Topic 5: Vaping is bad for youth | Concerns about the impact of vaping on youth, highlighting health risks and the prevalence of vaping in school environment. | vape, health, school, young, teen, child, issue, risk, high, problem | Vaping is the new smoking. Youth are developing nicotine addictions and lung health issues and big corporations are concealing the data! |
|  | Topic 6: Vaping is safer than smoking but not risk-free | Compares the harm of vaping to traditional smoking, arguing that it's a lesser evil, yet still acknowledging its risks. | vape, tobacco, smoking, cigarette, nicotine, smoker, smoke, adult, product, vaper | Ministers face calls to crack down on child vaping epidemic: Government-commissioned report rules trendy. e-cigs are 'very unlikely to be risk-free' even if they ARE safer than smoking ... |
|  | Topic 7: Urge to quit vaping due to health risks and addiction | Emphasizes the urgent need to quit vaping due to its association with serious health issues like cancer and addiction. | vaping, quit, cancer, smoking, disease, need, addicted, help, time, cause | I really need to quit smoking my juul. |
|  | Topic 8: Calls for quitting vaping due to health and social perceptions | Discusses the social judgments and stigmas associated with vaping, comparing it to other substance use like drinking or drug use. | vape, people, smoke, drink, drug, thing, weed, know, think, make | If you're drinking soda, smoking cigarettes, drinking in excess, vaping, or abusing drugs stop immediately!  Last night saw these people, drinking beer, vaping, smoking, and gambling. My mind is blown. |
|  | Topic 9: Dislike vaping, especially the waste coming from disposable vaping products. | This topic captures negative opinions about vaping. It focuses on individuals expressing their dislike for the habit and calling it a bad influence, particularly the growth of disposable vaping products and their implications. | vape, man, disposable, grow, get, make, old, year, ass, go | Disposable vapes/pods are sooo bad for the environment they’re like full on man-made material even worse than cigarette filters. |
